# Molecular imaging biomarkers in familial frontotemporal lobar degeneration: Progress and prospects

**DOI:** 10.3389/fneur.2022.933217

**Published:** 2022-08-16

**Authors:** Ruihan Wang, Hui Gao, Hongsheng Xie, Zhiyun Jia, Qin Chen

**Affiliations:** ^1^Department of Neurology, West China Hospital of Sichuan University, Chengdu, China; ^2^Department of Nuclear Medicine, West China Hospital of Sichuan University, Chengdu, China

**Keywords:** familial frontotemporal lobar degeneration, molecular imaging, biomarkers, MAPT, GRN, C9orf72

## Abstract

Familial frontotemporal lobar degeneration (FTLD) is a pathologically heterogeneous group of neurodegenerative diseases with diverse genotypes and clinical phenotypes. Three major mutations were reported in patients with familial FTLD, namely, progranulin (*GRN*), microtubule-associated protein tau (*MAPT*), and the chromosome 9 open reading frame 72 (*C9orf72*) repeat expansion, which could cause neurodegenerative pathological changes years before symptom onset. Noninvasive quantitative molecular imaging with PET or single-photon emission CT (SPECT) allows for selective visualization of the molecular targets *in vivo* to investigate brain metabolism, perfusion, neuroinflammation, and pathophysiological changes. There was increasing evidence that several molecular imaging biomarkers tend to serve as biomarkers to reveal the early brain abnormalities in familial FTLD. Tau-PET with ^18^F-flortaucipir and ^11^C-PBB3 demonstrated the elevated tau position in patients with FTLD and also showed the ability to differentiate patterns among the different subtypes of the mutations in familial FTLD. Furthermore, dopamine transporter imaging with the ^11^C-DOPA and ^11^C-CFT in PET and the ^123^I-FP-CIT in SPECT revealed the loss of dopaminergic neurons in the asymptomatic and symptomatic patients of familial FTLD. In addition, PET imaging with the ^11^C-MP4A has demonstrated reduced acetylcholinesterase (AChE) activity in patients with FTLD, while PET with the ^11^C-DAA1106 and ^11^C-PK11195 revealed an increased level of microglial activation associated with neuroinflammation even before the onset of symptoms in familial FTLD. ^18^F-fluorodeoxyglucose (FDG)-PET indicated hypometabolism in FTLD with different mutations preceded the atrophy on MRI. Identifying molecular imaging biomarkers for familial FTLD is important for the *in-vivo* assessment of underlying pathophysiological changes with disease progression and future disease-modifying therapy. We review the recent progress of molecular imaging in familial FTLD with focused on the possible implication of these techniques and their prospects in specific mutation types.

## Introduction

Frontotemporal lobar degeneration (FTLD) encompasses a set of clinical syndromes characterized by progressive abnormalities in behavior, executive function, language, or motor function. Patients with FTLD may present clinical syndromes with the behavioral variant of frontotemporal dementia (bvFTD), the nonfluent variant of a primary progressive aphasia (nfvPPA), a semantic variant of PPA (svPPA), and some patients also have amyotrophic lateral sclerosis (ALS), corticobasal syndrome (CBS) or progressive supranuclear palsy (PSP) (1). Approximately, 40% of patients with FTLD have a positive family history of autosomal dominant inheritance (2). Three major mutations were reported in patients with familial FTLD, namely, the microtubule-associated protein tau (*MAPT*), progranulin (*GRN*), and the repeat expansions in the chromosome 9 open reading frame 72 (*C9orf72*). These mutations could lead to neurodegenerative pathological changes years before symptom onset (2, 3).

Mutations in the *MAPT* gene located on chromosome 17q21 first reported in 1998 (4) were discovered in numerous pedigrees of familial FTLD. The majority of known mutations in the coding region occur in the repeats, causing the decreased ability of tau proteins to interact with microtubules, and resulting in hyperphosphorylated tau accumulation in neurons and glial cells (5). *MAPT* mutations of different subtypes have been linked to various tauopathies. Generally, the mutations inside exon 10 (i.e., N279K, S305N, P301L) and intron 10 (i.e., IVS 10 + 16) tend to form four tandem microtubule-binding domain repeat (4R-tau) pathology, while mutations outside exon 10 (i.e., V337M, R406W, Q351R) tend to form mixed 3R/4R tauopathy similar to the tauopathy in Alzheimer's disease (6).

Mutations in the *GRN* linked to chromosome 17q21 initially reported in 2006 (7, 8) could result in a lack of progranulin by haploinsufficiency and the accumulation of TAR DNA-binding protein (TDP)-43 protein (9). *GRN* mutation carriers have a wide range of clinical phenotypes and illness onset ages. The bvFTD, CBS, and PPA are the most common clinical syndromes in patients with *GRN* mutation (10, 11).

The hexanucleotide GGGGCC (G4C2) repeat expansions of the *C9orf72* were identified as a common genetic cause of FTLD and ALS in 2011 (12, 13). TDP-43 aggregations were pathologically discovered in cases with *C9orf72* expansions (14). The most prevalent clinical syndromes were bvFTD, ALS, or a mixture of both in *C9orf72* mutation carriers (14, 15).

Currently, the visual inspection of MRI was demonstrated to easily increase the diagnostic confidence of underlying FTLD (16, 17). The cortical microstructure was found to be more sensitive than cerebral atrophy within patients with GRN mutations (18), suggesting the powerful value of MRI to correctly diagnose and capture the early abnormalities in familial FTLD. Noninvasive quantitative molecular imaging with PET or single-photon emission CT (SPECT) provided another perspective and allowed for selective visualization of the molecular targets *in vivo* to investigate the brain topographic and pathophysiological changes. The former included metabolism, perfusion, neuroinflammation, synaptic function, and neurotransmitters' activity, and the latter comprised Tau and Aβ aggregation. There was increasing evidence that several molecular imaging biomarkers tend to serve as biomarkers to reveal the early brain abnormalities in familial FTLD. Identifying molecular imaging biomarkers for familial FTLD is important for the *in-vivo* assessment of underlying pathophysiological changes with disease progression and future disease-modifying therapy. Thus, we review the recent progress of molecular imaging in familial FTLD with a focus on the possible implication of these techniques and their prospects in specific mutation types.

## Methods

### Search strategy

We performed electronic searches of Medline, PubMed, and Embase databases using the combination of a number of medical subject headings, Emtree subject headings, and free-text terms (“frontotemporal lobar degeneration,” and “frontotemporal dementia” for clinical categories; “microtubule-associated protein tau” or “*MAPT*,” “progranulin” or “*GRN*,” and “chromosome 9 open reading frame 72” or “*C9orf72*” for genes; “positron emission tomography” or “PET,” “single-photon emission CT” or “SPECT,” and “dopamine transporter imaging” for molecular imaging biomarkers). The retrieval deadline was 1 December 2021. All the relevant articles were retrieved, placing restrictions on #elds (free-text terms searched exclusively in the title or abstract of the articles) and publication type (original articles).

## Discussion

### Pathophysiological biomarkers

#### Tau studies

Tau-PET is currently being explored as a promising method to identify the tau protein *in vivo* (19). Several types of tracers have been applied to map the pattern of tau accumulation in familial FTLD, especially in individuals with *MAPT* mutations thought to be tauopathy. ^18^F-flortaucipir, the most commonly used tau tracer, has been proven to bind paired helical filaments composed of 3R/4R tau in Alzheimer's disease (AD) (20, 21). In recent years, other tracers, including ^11^C-PBB3 (22), ^18^F-MK6240 (23), and ^18^F-PM-PBB3 (24), started to be applied in *MAPT* mutation carriers. ^11^C-PBB3 could capture wide-ranging Tau pathologies, including 3R/4R tau and 4R tau (25, 26) compared to^18^F-flortaucipir (27). For ^18^F-MK6240 and ^18^F-PM-PBB3, no clear off-target binding was reported with the improved design (28–31). ^18^F-PM-PBB3 has shown higher binding affinities to 4R tau compared with the 3R/4R tracer ^18^F-MK6240 (24). The novel tau tracers might help show diverse tau pathologies in various mutation subtypes.

##### *MAPT*_Tau-PET

Frontotemporal lobar degeneration with *MAPT* mutations is regarded as tauopathy (32), and tau PET provides an effective way to explore biomarkers for multiform tau pathologies in a homogeneous patient group (33). Most individuals with *MAPT* mutations inside exon 10 (i.e., P301L, S305N, N279K) and intron 10 (i.e., IVS 10 + 16) had 4R tau pathology, while with *MAPT* mutations outside exon 10 (i.e., V337M, R406W, Q351R) had 3R/4R paired helical filament tau pathology (34). *MAPT* mutations have different types of underlying tauopathies, leading to different tracer binding patterns.

^18^F-flortaucipir was most commonly used to track 3R/4R tau, so most participants with *MAPT* mutations outside exon 10 showed a higher-level tracer binding than mutations in exon 10 (34). Only a few ^18^F-flortaucipir studies included 4 asymptomatic *MAPT* mutation carriers. 3 of them (1 N279K, 1 R406W, and 1 IVS 10 + 16) had little to no uptake, but the other *MAPT* R406W mutation carrier had a signal in the AD range (34, 35). The heterogeneous results might be explained by quite a limited sample size. In symptomatic *MAPT* mutation carriers, temporal (36–42), insular (37, 41, 42), and frontal (38, 39, 41) regions were most commonly reported for increased ^18^F-flortaucipir uptake. Especially, in two *MAPT* R406W mutation carriers with short disease duration, the hippocampus and adjacent temporal lobe regions were mainly involved, whereas in another *MAPT* R406W mutation carrier with a long duration, tau aggregation spreads across the whole temporal, frontal lobes, and the basal ganglia (39). Moreover, a longitudinal study of a *MAPT* Q351R mutation carrier also demonstrated that tau aggregation expanded from the insula region cortically, and the medial temporal, putamen, and pallidum regions subcortically to the temporal region cortically and caudate and thalamus regions subcortically even just over 1 year (37). These findings suggested that ^18^F-flortaucipir might be a sensitive biomarker for disease progression in symptomatic *MAPT* mutation carriers. However, the majority of research was based on case reports or cross-sectional studies with small sample size. Longitudinal data with larger cohorts will be required for such investigations.

Two studies applied ^11^C/^18^F-PBB3 tracking both the 3R/4R tau and 4R tau in symptomatic *MAPT* mutation carriers (22, 24). In four patients with *MAPT* N279K mutation, the kindred with slow progression exhibited mild binding; in contrast, kindreds with rapid progression showed profoundly increased binding in widespread regions from an early disease stage (22). Recently, a study of ^18^F-MK-6240 in two asymptomatic *MAPT* P301L mutation carriers showed modest tau deposition about 5 years from estimated onset (23), indicating that ^18^F-MK-6240 uptake might be an early biomarker for *MAPT* P301L mutation carriers (Table 1).

**Table 1:** Studies investigating *MAPT* mutation vs. controls.

**No**.	**References**	**No. of subjects**	**Techniques**	**Findings**
1	Arvanitakis et al. (43)	(2 a*MAPT*+, 5 s*MAPT*+) vs. 3 NC	^18^F-FDG PET	Asymmetric temporal lobe hypometabolism in 7 *MAPT*+
2	Laws et al. (44)	(31 s*MAPT*_H1+ 10s *MAPT*_H2) vs. 16 HC	^18^F-FDG PET	More pronounced hypometabolism in frontal brain areas of H2 carriers than H1 carriers
3	Deters et al. (45)	(3 a*MAPT*+, 8 s*MAPT*+) vs. 8 NC	^18^F-FDG PET	Hypometabolism bilaterally in the medial temporal lobe, and the parietal and frontal cortices
4	Yang et al. (46)	2 s*MAPT*+ vs. 1 NC	^18^F-FDG PET	Hypometabolism in extensive prefrontal areas, and hypermetabolism in the putamen, globus pallidum, cerebellum, and sensorimotor cortex
5	Su et al. (24)	1 s*MAPT*+ vs. HC	^18^F-FDG PET	Brain metabolism significantly decreased in bilateral temporal lobes and moderately decreased in bilateral frontal lobes with more remarkable in the left side
6	Clarke et al. (47)	6 a*MAPT*+ vs. 12 NC	^18^F-FDG PET	Hypometabolism in the anterior cingulate
7	Bevan Jones et al. (36)	1 s*MAPT*+ vs. 12 HC	^18^F-flortaucipir PET	Increased tau accumulation in the anterior temporal lobes and ventral anterior cingulate cortex
8	Smith et al. (39)	3 s*MAPT*+ vs. 4 HC	^18^F-flortaucipir PET	Increased tau accumulation mainly in the hippocampus and adjacent temporal lobe regions of 2 s*MAPT*+ with short disease duration and isolated memory impairment; the temporal, frontal lobes, and basal ganglia of 1 s*MAPT*+ with long disease duration and behavioral deficits
9	Spina et al. (41)	1 s*MAPT*+ vs. 20 HC	^18^F-flortaucipir PET	Increased tau accumulation in the bilateral frontal pole, medial orbitofrontal cortex, inferior temporal lobe, insular cortex, anterior cingulate, dorsolateral prefrontal cortex, and lateral temporal cortex
10	Jones et al. (34)	(3 a*MAPT*+, 10 s*MAPT*+) vs. 241 HC vs. 30 AD	^18^F-flortaucipir PET	The greatest tau accumulation in AD and minimal regional tau accumulation in *MAPT*+ with mutations in exon 10
11	Bevan Jones et al. (35)	1 a*MAPT*+ vs. 13 HC	^18^F-flortaucipir PET	A lack of tau aggregation in frontotemporal regions
12	Tsai et al. (42)	6 s*MAPT*+ vs. 53 HC	^18^F-flortaucipir PET	Tau depositions in left insula and bilateral temporal poles
13	Convery et al. (37)	1 s*MAPT*+ vs. 6 HC	^18^F-flortaucipir PET	Baseline: tau aggregation in the insula region cortically, and the medial temporal, putamen, and pallidum regions subcortically Follow-up: tau aggregation in the same regions as at baseline but also the temporal region cortically and caudate and thalamus regions subcortically
14	Soleimani-Meigooni et al. (40)	2 s*MAPT*+ vs. 14 HC	^18^F-flortaucipir PET	Tau depositions in the temporal lobes, temporal white matter, and basal ganglia
15	Malpetti et al. (48)	2 s*MAPT*+ vs. 15 HC	^18^F-flortaucipir PET	Consistent tau deposition distribution in frontotemporal regions in 2 s*MAPT*+
16	Ikeda et al. (22)	4 s*MAPT*+ vs. 13 HC	^11^C-PBB3 PET	Mild tau depositions in the midbrain and medial temporal areas of 2 s*MAPT*+ from kindred with slow progression; profoundly increased tau depositions in widespread regions of 2 s*MAPT*+ from kindreds with rapid progression
17	Su et al. (24)	1 s*MAPT*+ vs. HC	^18^F-PM-PBB3 PET	Slightly diffuse tau deposition especially in the left frontal lobe
18	Levy et al. (23)	(3 a*MAPT*+, 3 s*MAPT*+) vs. 83 HC	^18^F-MK-6240 PET	At least mild but significant tau deposition in 3 s*MAPT*+; modest tau deposition in 2 a*MAPT*+ within 5 years from estimated onset; no tau deposition in 1 a*MAPT*+ about 30 years from estimated onset
19	Miyoshi et al. (49)	3 aMAPT+ vs. 9 HC	^11^C-DOPA PET	Low dopamine synthesis in putamen
20	Yang et al. (46)	2 s*MAPT*+ vs. 1 NC	^11^C-CFT PET	Dopaminergic dysfunction in the caudate nucleus and putamen
21	Wu et al. (50)	(3 a*MAPT*+, 1 s*MAPT*+) vs. 6 HC	^11^C-CFT PET	Dopaminergic dysfunction is severe in s*MAPT*+ and mild in a*MAPT*+
22	Smith et al. (39)	3 s*MAPT*+ vs. 4 HC	Amyloid-PET (^18^F-flutemetamol)	Negative in all participants
23	Tsai et al. (42)	5 s*MAPT*+ vs. 53 HC	Amyloid-PET (^11^C-PiB)	Positive in 1 s*MAPT*+
24	Soleimani-Meigooni et al. (40)	2 s*MAPT*+ vs. 14 HC	Amyloid-PET (^11^C-PiB)	Positive in 1 s*MAPT*+
25	Su et al. (24)	1 s*MAPT*+ vs. HC	Amyloid-PET (^18^F-florbetapir)	Negative in 1 s*MAPT*+
26	Levy et al. (23)	(3 a*MAPT*+, 3 s*MAPT*+) vs. 83 HC	Amyloid-PET (^18^F-flutafuranol)	Negative in all participants
27	Seelaar et al. (51)	10 s*MAPT*+ vs. 10 HC	^99m^Tc-HMPAO SPECT	Hypoperfusion in the left temporal and inferior frontal gyri
28	Chaunu et al. (52)	1 s*MAPT*+	^99m^Tc-HMPAO SPECT	Hypoperfusion in the bilateral left predominant frontotemporal and basal ganglia
29	Miyoshi et al. (49)	3 a*MAPT*+ vs. 9 HC	^11^C-DAA1106 PET	Increased Glial activities in the frontal cortex of 1 a*MAPT*+, the occipital cortex of 2 a*MAPT*+, and the posterior cingulate cortex of 1 a*MAPT*+
30	Bevan-Jones et al. (35)	1 a*MAPT*+ vs. 15 HC	^11^C-PK11195 PET	Microglial activation in frontotemporal regions
31	Malpetti et al. (48)	2 s*MAPT*+ vs. 15 HC	^11^C-PK11195 PET	Tau deposition overlapped with that of microglial activation but more extensive
32	Miyoshi et al. (49)	3 a*MAPT*+ vs. 9 HC	^11^C-MP4A PET	Decreased AChE activity in the temporal, parietal cortex

*aMAPT+, asymptomatic MAPT mutation carriers; sMAPT+, symptomatic MAPT mutation carriers; HC, healthy controls; NC, non-carriers; FDG, fluorodeoxyglucose; HMPAO, hexamethylpropylene amine oxime; PiB, Pittsburgh compound B; PET, positron emission tomography; SPECT, single photon emission computed tomography; AChE, acetylcholinesterase*.

In *MAPT* mutation carriers, the value of tau PET for capturing tau accumulation has been primarily proved, and the tau aggregation patterns were associated with the subtypes of mutations and tracers. Therefore, novel tracers for multiform tau pathologies need to be further explored in longitudinal studies with larger cohorts.

##### *GRN*/*C9*orf72_Tau-PET

Three studies reported ^18^F-flortaucipir binding in the frontotemporal region in five symptomatic *GRN* mutation carriers (38, 40, 42), whereas another research found no ^18^F-flortaucipir binding in a patient with *GRN* mutation (53) (Table 2). Similarly, findings among symptomatic *C9orf72* mutation carriers were contradictory. Ten patients with *C9orf72* mutation had increased ^18^F-flortaucipir binding in the frontal lobe (38, 40, 42, 64), while another study found no tau deposition in six patients with *C9orf72* mutation (65) (Table 3). The contradictory results might be associated with the small number of participants and different clinical phenotypes. Moreover, a study showed that ^18^F-MK-6240 PET scan was negative for three individuals with *GRN* or *C9orf7*2 mutations (23), implying that ^18^F-MK-6240 might not be an optimal method for tracking tau deposition in *GRN* or *C9orf72* mutation carriers.

**Table 2:** Studies investigated asymptomatic/symptomatic *GRN* carriers.

**No**.	**References**	**No. of subjects**	**Techniques**	**Findings**
1	Huey et al. (54)	2 s*GRN*+	^18^F-FDG PET	Predominant right-sided hypometabolism
2	Jacova et al. (55)	9 *GRN*+ (4 a*GRN*+) vs. 11 NC (8 aNC)	^18^F-FDG PET	*GRN*+ showed an overall pattern of right anterior cerebral hypometabolism
3	Josephs et al. (56)	3 s*GRN*+ vs. 3 sNC vs. 26 HC	^18^F-FDG PET	s*GRN*+ and sNC vs. HC: left temporoparietal hypometabolism s*GRN*+ vs. sNC: more severe anteromedial temporal hypometabolism
4	Caroppo et al. (57)	Baseline: 16 a*GRN*+ VS 17 NC Follow-up: 14 a*GRN*+ VS 14 NC	^18^F-FDG PET	Baseline: left middle temporal gyrus hypometabolism Follow-up: left inferior temporal, left middle frontal, left inferior orbital frontal, right superior orbital frontal gyri, left thalamus hypometabolism
5	Licata et al. (58)	10 s*GRN*+ vs. 23 HC	^18^F-FDG PET	Inter-individual variability of FDG uptake pattern in s*GRN*+. All s*GRN*+ showed frontal hypometabolism. Asymmetrical metabolism in half of s*GRN*+
6	Deng et al. (59)	1 s*GRN*+	^18^F-FDG PET	Bifrontal and bitemporal hypometabolism
7	Ljubenkov et al. (60)	26 *GRN*+ (18 s*GRN*+) vs. 52 HC	^18^F-FDG PET	Left-predominant hypometabolism in dorsal prefrontal, anterior cingulate, orbitofrontal, inferior frontal gyrus, insular, lateral parietal, lateral temporal, posterior cingulate, caudate, and thalamic regions Bifrontal hypometabolism was associated with worse clinical symptoms rating
8	Lagarde et al. (53)	1 s*GRN*+ vs. 8 sporadic FTLD	^18^F-flortaucipir PET	No tau binding in s*GRN*+; tau binding in 5/8 sNC
9	Carecchio et al. (61)	1 s*GRN*+	DaTScan (^123^I-ioflupane SPECT)	Reduced tracer uptake in the left putamen
10	Deng et al. (59)	1 s*GRN*+	^18^F-DOPA PET	^18^F-DOPA: reduced DOPA metabolism in bilateral corpus striatum
11	Josephs et al. (56)	3 s*GRN*+ vs. 3 sporadic FTLD vs. 26 HC	Amyloid-PET (^11^C-PiB)	Negative in all participants (cut-off score of <1.5). s*GRN*+ had lower PiB-PET ratios compared to sNC
12	Dopper et al. (62)	1 s*GRN*+	^99m^Tc-HMPAO SPECT	Symmetrical frontoparietal hypoperfusion.
13	Premi et al. (63)	13 s*GRN*+ vs. 13 sporadic FTLD vs. 13 HC	^99m^Tc-ECD SPECT	s*GRN*+ and sNC vs. HC: hypoperfusion in frontotemporal areas s*GRN*+ vs. sNC: hypoperfusion in anterior cingulate cortex and left dorsolateral prefrontal cortex
14	Carecchio et al. (61)	1 s*GRN*+	perfusion SPECT	Left predominant bifrontal with homolateral parieto-temporal hypoperfusion

*GRN+, GRN mutation carriers; NC, non-carriers; HC, healthy controls; GRN+, symptomatic GRN mutation carriers; aGRN+, asymptomatic GRN mutation carriers; FDG, fluorodeoxyglucose; ECD, ethylcysteinate dimer; HMPAO, hexamethylpropylene amine oxime; PiB, Pittsburgh compound B; DaTscan, dopamine transporter scan; PET, positron emission tomography; SPECT, single photon emission computed tomography*.

**Table 3:** Studies investigated asymptomatic/symptomatic *C9* carriers.

**No**.	**References**	**No. of subjects**	**Techniques**	**Findings**
1	Gramaglia et al. (66)	1 s*C9*+	^18^F-FDG PET	Bilateral frontotemporal hypometabolism
2	Martikainen et al. (67)	1 s*C9*+	^18^F-FDG PET	Hypometabolism in temporal lobes
3	Solje et al. (68)	36 s*C9*+	^18^F-FDG PET	Normal in 17.6% of s*C9*+
4	Block et al. (69)	1 s*C9*+	^18^F-FDG PET	Symmetric and mild medial-greater-than-lateral bifrontal hypometabolism
5	Sha et al. (70)	1 s*C9*+	^18^F-FDG PET	Bilateral frontal and temporoparietal hypometabolism
6	Castelnovo et al. (71)	9 s*C9*+	^18^F-FDG PET	Prevalent frontal hypometabolism in bvFTD *C9*+ Right temporal polar and lateral hypometabolism in svPPA *C9*+
7	Diehl-Schmid et al. (72)	22 s*C9*+ vs. 22 sporadic FTLD vs. 23 HC	^18^F-FDG PET	s*C9*+ vs. sNC: a significant reduction of glucose metabolism in both thalami
8	Levy et al. (73)	1 s*C9*+	^18^F-FDG PET	Bifrontal hypermetabolism; no significant areas of hypometabolism
9	Sellami et al. (74)	1 s*C9*+	^18^F-FDG PET	Bilateral frontal and anterior temporal hypometabolism
10	De Vocht et al. (75)	17 a*C9*+ vs. 25 HC	^18^F-FDG PET	Hypometabolism in frontotemporal regions, basal ganglia, and thalami of a*C9*+
11	Filikci et al. (76)	1 s*C9*+	^18^F-FDG PET	Hypometabolism in parietotemporal cortex, posterior cingulate gyrus and precuneus, mesial temporal lobes, and frontal lobes
12	Popuri et al. (77)	15 a*C9*+ vs. 20 NC	^18^F-FDG PET	Cingulate gyrus, frontal, and temporal neocortices (left >right) and bilateral thalami hypometabolism
13	Bevan-Jones et al. (64)	1 s*C9*+ vs. 13 NC	^18^F-flortaucipir PET	Increased binding in frontotemporal cortex of sym *C9*+
14	Smith et al. (65)	6 s*C9*+ vs. 6 sv PPA vs. 54 HC	^18^F-flortaucipir PET	*C9*+ exhibited none or limited ^18^F-flortaucipir retention
15	Filikci et al. (76)	1 s*C9*+	DaTScan	Unremarkable DaTscan
16	Martikainen et al. (67)	1 s*C9*+	Amyloid-PET (^11^C-PiB)	Negative amyloid PET
17	Block et al. (69)	1 s*C9*+	Amyloid-PET	Negative amyloid PET.
18	Sha et al. (70)	1 s*C9*+	Amyloid-PET (^11^C-PiB)	Positive amyloid PET.
19	Filikci et al. (76)	1 s*C9*+	Amyloid PET (^11^C-PiB)	Negative amyloid PET
20	Malpetti et al. (48)	3 a*C9*+ vs. 1 sporadic FTLD vs. 19 HC	^11^C-UCB-J PET	a*C9*+ vs. HC: reduced synaptic density in the thalamus

*C9+, C9orf72 mutation carriers; NC, non-carriers; HC, healthy controls; sC9+, symptomatic C9orf72 mutation carriers; aC9+, asymptomatic C9orf72 mutation carriers; bvFTD, behavioral variant frontotemporal dementia; svPPA, semantic variant primary progressive aphasia; FDG, fluorodeoxyglucose; PiB, Pittsburgh compound B; DaTscan, dopamine transporter scan; PET, positron emission tomography; SPECT, single photon emission computed tomography*.

#### Amyloid studies

To detect the underlying AD pathology, amyloid-PET with tracers, including ^11^C-Pittsburgh compound B (PiB) (42, 67, 70, 76), ^18^F-florbetapir (24), ^18^F-florbetaben (23), ^18^F-flutafuranol (78), ^18^F-flutemetamol (39, 79), etc., is applied in patients with familial FTLD.

Most patients with *MAPT* mutation indicated negative results with ^11^C-PiB or ^18^F-florbetapir PET (23, 24, 39, 42), while two patients with *MAPT* P301L mutation had a positive ^11^C-PiB scan (40, 42). However, one might imply an incidental rather than preclinical β-amyloid pathology since the SUVRs were well below those seen in AD (42); in contrast, the other regarded as combining with AD presented higher SUVRs close to AD (40). Negative results with ^11^C-PiB or ^18^F-flutafuranol were reported in patients with *GRN* and *C9orf72* mutation carriers so far (23, 42, 56, 76). Thus, amyloid-PET may help discriminate true underlying AD co-pathology from incidental β-amyloid pathology (80) (Table 4).

**Table 4:** Studies investigating multiple different mutations in FTLD.

**No**.	**References**		**No. of subjects**	**Techniques**	**Findings**
1	Tsai et al. (42)		6 s*MAPT*+ vs. 5 s*C9*+ vs. 1 s*GRN*+ vs. 53 HC	^18^F-flortaucipir PET	Tau deposition in the left insula and bilateral temporal poles of s*MAPT*+; the left lateral frontal, parietal and temporal lobes of s*GRN*+; the frontal poles of s*C9*+ with varying degrees
2	Soleimani-Meigooni et al. (40)		2 s*MAPT*+ vs. 1 s*C9*+ vs. 1 s*GRN*+ vs. 14 HC	^18^F-flortaucipir PET	Tau deposition was less than Alzheimer's disease, though higher than HC, and did not reliably correspond with post-mortem tau pathology for all mutation groups
3	Malpetti et al. (48)		2 s*MAPT*+ vs. 3 s*C9*+ vs. 2 s*GRN*+ vs. 15 HC	^18^F-flortaucipir PET	Consistent tau deposition distribution (overlapped with that of ^11^C-PK11195, but was more extensive) in 2 s*MAPT*+, heterogeneous tau deposition distributions among s*GRN*+ and s*C9*+
4	Levy et al. (23)		(3 a*MAPT*+, 3 s*MAPT*+) vs. 2 s*C9*+ vs. 2 s*GRN*+ vs. 83 HC	^18^F-MK-6240 PET	At least mild but significant tau deposition in 3 s*MAPT*+; modest tau deposition in 2 a*MAPT*+ within 5 years from estimated onset; no tau deposition in 1 a*MAPT*+ about 30 years from estimated onset Negative for 2 s*GRN*+, and 1 advanced s*C9*+ showed minimal regionally non-specific binding
5	Tsai et al. (42)		5 s*MAPT*+ vs. 4 s*C9*+ vs. 1 s*GRN*+ vs. 53 HC	Amyloid-PET (^11^C-PiB)	Positive in 1 s*MAPT*+ and 1 s*GRN*+
6	Levy et al. (23)		(3 a*MAPT*+, 3 s*MAPT*+) vs. 2 s*C9*+ vs. 2 s*GRN*+ vs. 83 HC	Amyloid-PET (^18^F-flutafuranol)	Negative in all participants
7	Seelaar et al. (51)		10 s*MAPT*+ vs. 19 FTLD-TDP (6 *GRN*+, 5 Ser82ValfsX174+, 1 Gln125X+, 13 unknown gene defect) vs. 10 HC	^99m^Tc-HMPAO SPECT	Hypoperfusion in the right frontal lobe, precuneus, cuneus, and inferior parietal lobule of familial FTLD-TDP; in the left temporal and inferior frontal gyri of *MAPT*+
8	Lant et al. (81)		10 s*MAPT*+ vs. 9 s*C9*+ vs. 8 s*GRN*+ vs. 13 AD vs. 13 HC	^11^C-PK11195 PET	Significantly microglial activation in all four regions (cortical gray and subcortical white matter of frontal and temporal) of FTLD Greater microglial activation of frontal subcortical white matter in FTLD than AD, temporal cortical gray matter in contrast Microglial activation was higher in FTLD-*MAPT* than other genetic forms (*GRN, C9*)
9	Malpetti et al. (48)		2 s*MAPT*+ vs. 3 s*C9*+ vs. 2 s*GRN*+ vs. 15 HC	^11^C-PK11195 PET	Increased microglial activation predominantly in frontotemporal regions for all mutation groups

*FTLD, frontotemporal lobar degeneration; TDP, TAR DNA binding protein; aMAPT+, asymptomatic MAPT mutation carriers; sMAPT+, symptomatic MAPT mutation carriers; sC9+, symptomatic C9orf72 mutation carriers; sGRN+, symptomatic GRN mutation carriers; HC, healthy controls; NC, non-carriers; HMPAO, hexamethylpropylene amine oxime; PiB, Pittsburgh compound B; PET, positron emission tomography; SPECT, single photon emission computed tomography*.

### Topographic biomarkers

#### Brain metabolism

^18^F-fluorodeoxyglucose (FDG)-PET is a technique for measuring glucose metabolism *in vivo* (82). Studies of FDG-PET could capture the different patterns of brain hypometabolism and even precede brain atrophy in familial FTLD mutation carriers (43, 45, 47, 55, 57, 72, 83).

##### *MAPT*_FDG-PET

A few cross-sectional FDG-PET studies demonstrated brain hypometabolism in both the asymptomatic and symptomatic *MAPT* mutation carriers (24, 43, 45–47). Hypometabolism in the temporal lobe (43, 45) and anterior cingulate cortex (47) was reported in asymptomatic *MAPT* mutation carriers, while temporal lobe hypometabolism even preceded the brain atrophy on MRI in the asymptomatic stage (43). In symptomatic *MAPT* mutation carriers, hypometabolism regions spread extensively to the frontotemporal lobes (24, 43, 46), while hypermetabolism was also found in the putamen, globus pallidum, cerebellum, and sensorimotor cortex (46). These findings all pointed to early involvement of the temporal lobe in asymptomatic *MAPT* mutation carriers. Furthermore, only one study compared three asymptomatic *MAPT* mutation carriers and 8 symptomatic *MAPT* mutation carriers, but found no difference in FDG uptake (45), which was mainly due to the small sample size. However, most current studies were cross-sectional with a small cohort, and further studies are needed to characterize the trajectories of metabolism patterns from asymptomatic to symptomatic *MAPT* mutation carriers.

##### *GRN*_FDG-PET

Two studies indicated asymmetric temporal lobe hypometabolism with FDG-PET in asymptomatic *GRN* mutation carriers (55, 57). After 20 months of follow-up, hypometabolism spread to the frontal lobe and thalamus (57). The metabolic changes appeared before brain atrophy on MRI and approximately more than 10 years before clinical onset (57), suggesting that FDG-PET changes can be detected as early biomarkers in *GRN* mutation carriers. In symptomatic *GRN* mutation carriers, the asymmetrical hypometabolism of temporoparietal (56) and frontal (58) lobes was reported primarily based on a small number of cross-sectional studies or case reports. Hypometabolism patterns were observed to correlate with clinical manifestations (56), but another study failed to find clear metabolic change pattern in each clinical subtype (58).

##### *C9orf72*_FDG-PET

In asymptomatic *C9orf72* mutation carriers, extensive hypometabolism was observed in frontotemporal and subcortical regions in two studies (75, 77). Thalami hypometabolism was found in both the asymptomatic (75, 77) and symptomatic (72) individuals with *C9orf72* mutation, especially when compared to sporadic FTLD patients (72), suggesting that thalami could be a distinguishing early biomarker for *C9orf72* mutation carriers. In symptomatic *C9orf72* mutation carriers, some studies showed that the hypometabolism patterns were consistent with the clinical diagnosis and correlated well with the brain atrophy on MRI, for example, prevalent frontal hypometabolism in patients with bvFTD and temporal polar and lateral temporal hypometabolism in patient with svPPA (66, 69, 71, 74). However, the cross-sectional studies above with small sample sizes still need to be replicated in longitudinal studies with larger cohorts.

Most studies demonstrated the concordance between structural MRI and FDG-PET in *MAPT* (43, 45), *GRN* (84, 85), and *C9orf72* (74, 77) mutation carriers. However, controversy still existed regarding the earlier or more sensitive biomarkers (43, 45, 77). Some studies showed that additional informative MRI modalities such as diffusion tensor imaging (DTI) and arterial spin labeling (ASL) had equivalent or even better diagnostic utility of FTLD compared with FDG-PET (86–89), but others found a gap in sensitivity or accuracy that still remained (90, 91). Further investigations of familial FTLD need to compare the clinical value of microstructural MRI and PET.

#### Dopaminergic system

Dopamine functional deficits can be measured *in vivo via* PET or SPECT with various types of tracers assessing dopamine synthesis and storage [^18^F-DOPA, ^11^C-DOPA, ^11^C-dihydrotetrabenazine (DTBZ), ^18^F-fluoropropyl-DTBZ, etc.], transporter density (^123^I-FP-CIT, ^123^I-ioflupane,^11^C-CFT, ^99m^Tc-TRODAT, etc.), or postsynaptic terminals [^11^C-raclopride, ^123^I-iodobenzamide (IBZM), etc.] (92). Dopaminergic deficits were evaluated by the techniques mentioned above, especially in patients with familial FTLD with Parkinsonism.

Parkinsonism may present as the initial symptom in *MAPT* mutation carriers, particularly individuals with *MAPT* N279K mutation. Tracers such as ^11^C-DOPA and 2b-carbomethoxy-3b-(4-trmethylstannylphenyl) tropane (^11^C-CFT) were used to reveal dopaminergic function. The ^11^C-CFT uptake in the putamen was mildly low in asymptomatic *MAPT* N279K mutation carriers (49, 50). In symptomatic patients, both the caudate nucleus and putamen were involved more heavily (46, 50).

Individuals with *GRN* mutations and Parkinsonism could show reduced DOPA metabolism in bilateral corpus striatum by ^18^F-DOPA PET (59) or reduced tracer uptake in left putamen by ^123^I-ioflupane SPECT (61). Parkinsonism is not uncommon in *GRN* mutation carriers and sporadic patients with FTLD.

#### Brain perfusion

Perfusion SPECT is a well-established technique for measuring regional cerebral blood flow (rCBF) to assess brain function (93). The tracers utilized in brain perfusion SPECT are technetium-99m-hexamethylpropyleneamineoxime (^99m^Tc-HMPAO) and technetium-99m-ethylcysteinate dimer (^99m^Tc-ECD), both which are distributed proportionally to rCBF (93). Perfusion imaging has been widely used in the clinical evaluation of patients with neurological and psychiatric diseases (94), including FTLD.

In 11 *MAPT* mutation carriers, including eight in P301L, two in G272V, and one in G389R, significant hypoperfusion detected by ^99m^Tc-HMPAO SPECT was found in the asymmetric frontotemporal lobes (51, 52). Several studies indicated that hypoperfusion occurred in frontal areas of *GRN* mutation carriers (61–63). Compared with *MAPT* mutation carriers, patients with *GRN* mutation exhibited relatively more posterior hypoperfusion, including the precuneus and inferior parietal lobule detected by ^99m^Tc-HMPAO SPECT (51). Perfusion SPECT might be a potential biomarker to identify *MAPT* and *GRN* mutation carriers.

#### Neuroinflammation

Previous studies of genome-wide association (95) and animal (96) suggest that neuroinflammation might be an earlier process in FTLD, even preceding tau accumulation. The neuroinflammation is accompanied by the activation of microglia, and 18 kDa TSPO, previously known as peripheral benzodiazepine receptors, is highly expressed (97). Thus, radioligands (^11^C-PK11195, ^11^C-DAA1106) have been developed to target TSPO to visualize neuroinflammation *in vivo* (98, 99).

In asymptomatic *MAPT* mutation carriers, two studies with ^11^C-PK11195 PET (35) or ^11^C-DAA1106 PET (49) revealed increased levels of microglial activation, even despite a lack of significant atrophy or ^18^F-flortaucipir uptake (35). In symptomatic patients, ^18^F-flortaucipir binding overlapped with ^11^C-PK11195 binding and was more extensive across the brain (38). These findings suggest that neuroinflammation might facilitate tau aggregation initially, then tau-mediated neurodegeneration takes the dominant role. Combining different modalities in a relatively homogeneous group such as familial FTLD with a specific mutation subtype would better understand the underlying mechanism of disease progression.

Across different mutation subtypes, familial patients with FTLD with *MAPT, GRN*, and *C9orf7*2 mutations all showed increased ^11^C-PK11195 binding predominantly in frontotemporal regions (38), and ^11^C-PK11195 binding was significantly higher in temporal subcortical white matter in *MAPT* mutation carriers than in other genetic (*GRN, C9orf7*2) mutation carriers or sporadic FTLD (81). Future studies could add more details to the neuroinflammation patterns of subtypes of familial FTLD.

#### Synaptic function and acetylcholinesterase activity

The synaptic vesicle glycoprotein 2A (SV2A) is a transmembrane protein ubiquitously expressed in secretory vesicles of synapsis in all the brain areas (100). It is critical for synaptic function (101), and it has been related to neurologic disorders such as AD and epilepsy (102–104). The density of SV2A could be quantified by the newly developed tracer ^11^C-UCB-J (105). Reduced synaptic density in the thalamus detected by ^11^C-UCB-J was found in three asymptomatic *C9orf72* mutation carriers compared to healthy controls. It proved the role of the thalamus in *C9orf72* mutation carriers again, especially before symptom onset (48). There is a lack of studies on synaptic density mapping in other early staged mutation carriers. Thus, its value and correspondence with other imaging techniques remain unknown.

^11^C-MP4A PET could reflect acetylcholinesterase (AChE) activity *in vivo*. A study showed reduced AchE activity in the temporoparietal cortex in one of three asymptomatic *MAPT* N279K mutation carriers (49). Therefore, more studies with larger sample sizes are needed to provide further evidence for ^11^C-MP4A PET in familial FTLD.

### Challenges and limitations of molecular imaging

Even though more and more tracers were approved by the US Food and Drug Administration and by the European Medicines Agency for clinical usage (106), the higher cost and longer acquisition times compared to MRI might limit the wide applications in clinical practice (107). Changes in the levels of human fluid components could reflect underlying pathophysiological processes, and several fluid biomarkers were available or showed potential values such as Aβ, tau, NfL, and progranulin. A lack of multicenter standardization of procedures and quality control would compromise the stability and reliability of outcomes (108). By contrast, molecular imaging could provide more robust and comprehensive (quantitative and spatial distribution) information. However, the unspecific binding was still a challenge. Off-target binding of first-generation tau tracers such as ^18^F-flortaucipir might interfere with the quantification in several brain regions (109). Further development of 4R tau and TDP-43 specific tracers was needed to move toward precise diagnoses in FTLD. Several studies demonstrated that some molecular imaging biomarkers of FTLD with mutations could be different from sporadic individuals (72, 81), suggesting findings in genetic FTLD that may not translate to sporadic FTLD.

## Conclusion

This review summarized recent molecular imaging findings in familial frontotemporal lobar degeneration regarding common genetic mutations. The application of advanced neuroimaging techniques in monogenetic familial FTLD provides a unique opportunity to study specific proteinopathies and their clinical phenotypes. Although various study designs and data analysis methods generated heterogeneous nonspecific results, some key biomarkers could still be identified, pointing to specific brain regions worth further exploring. The combination of multimodal neuroimaging would also help identify the underlying mechanism of these biomarkers. To date, this research topic has been limited by a large multicenter longitudinal cohort study and a comparison between asymptomatic/symptomatic mutation carriers and sporadic patients with FTLD. Thus, the changes in different time points of these biomarkers between FTLD mutation carriers and sporadic ones are largely unknown, and the prognostic value of these biomarkers is still unclear. Future studies could focus on these issues and provide more insight into the significance of these molecular imaging methods and their findings.

## Author contributions

RW contributed to data collection, analysis and interpretation of the data, and drafting of the manuscript. HG contributed to analysis and interpretation of the data and drafting of the manuscript. HX and ZJ revised the manuscript. QC contributed to design the study, interpretation of the data, and revised the manuscript. All authors contributed to the article and approved the submitted version.

## Funding

This work was funded by National Natural Science Foundation of China (82071203), Science and Technology Innovation 2030 Major Projects (2022ZD0213600), and Natural Science Foundation of Sichuan (2022NSFSC1325).

## Conflict of interest

The authors declare that the research was conducted in the absence of any commercial or financial relationships that could be construed as a potential conflict of interest.

## Publisher's note

All claims expressed in this article are solely those of the authors and do not necessarily represent those of their affiliated organizations, or those of the publisher, the editors and the reviewers. Any product that may be evaluated in this article, or claim that may be made by its manufacturer, is not guaranteed or endorsed by the publisher.
